# The prevalence and distribution of non-communicable diseases and their risk factors in Kasese district, Uganda

**DOI:** 10.5830/CVJA-2012-081

**Published:** 2013-04

**Authors:** Charles Kiiza Mondo, Marcel Andrew Otim, Robert Musoke, George Akol, Jackson Orem

**Affiliations:** Department of Medicine, College of Health Sciences, Makerere University, Kampala, Uganda; Department of Medicine, College of Health Sciences, Makerere University, Kampala, Uganda; Department of Medicine, College of Health Sciences, Makerere University, Kampala, Uganda; Alcomed Specialist Diagnostic Service, Kasese, Uganda; Uganda Cancer Institute and Department of Medicine, College of Health Sciences, Makerere University, Kampala, Uganda

**Keywords:** non-communicable diseases, WHO STEPs, smoking, obesity, physical activity

## Abstract

**Background:**

To date there has been no population-based survey of the major risk factors for non-communicable diseases (NCD) in Uganda. Hospital-based data from urban centres report an increasing burden of NCDs in Uganda. This population-based survey aimed to describe the prevalence of risk factors for NCDs in a rural Ugandan district.

**Methods:**

The survey was conducted using the WHO STEPwise approach to surveillance of non-communicable diseases (STEPS) methodology. Participants (*n* = 611) were residents of the Kasese district selected in a one-step, complete survey of a rural district. Standardised international protocols were used to record history of disease, and measure behavioural risk factors (smoking, alcohol consumption, fruit and vegetable consumption, physical activity), physical characteristics [weight, height, waist and hip circumferences, blood pressure (BP)], fasting blood glucose (BG) and total cholesterol (TC) levels. Data were analysed using simple descriptive analysis.

**Results:**

In this sample, the prevalence of hypertension (systolic BP ≥ 140 mmHg and/or diastolic BP ≥ 90 mmHg) was 22.1% for men and 20.5% for women. Fifteen per cent of men and 16.8% of women were overweight [body mass index (BMI) ≥ 25 kg/m^2^] and 4.9% of men and 9.0% of women were obese (BMI ≥ 30 kg/m^2^). Nine per cent of participants were diabetic, 7.2% ate five or more combined servings of fruit per day while only 1.2% ate five or more combined servings of vegetables per day. Fifty-one per cent of the population were physically inactive and 9.6% were daily smokers. Thirty-one per cent of females had fasting blood sugar levels (FBS) ≥ 6.1 mmol/l while 10% of males had FBS > 6.1 mmol/l.

**Conclusion:**

This study presents evidence on the magnitude of NCDs, their risk factors and gender distribution in a rural population in Uganda, a poor country in east-central Africa. These data, when combined with urban population data, could be useful in the formulation and advocacy of NCD policy and plans of action in Uganda.

## Abstract

Non-communicable diseases (NCDs) are currently responsible for 35% of all deaths in low- and middle-income countries,^1^ and this alarming figure is predicted to rise in the near future. The World Health Organisation projects that the burden of disease due to NCDs will increase rapidly in the years ahead. From a projected total of 58 million deaths from all causes in 2005, it was estimated that NCDs would account for 35 million deaths, which was double the number of deaths from all communicable diseases (including HIV/AIDS, tuberculosis and malaria), maternal and perinatal conditions and nutritional deficiencies combined.^1^

This epidemiological transition in global health from infectious diseases to NCDs is posing not only a threat to the health of those affected but also places an enormous burden on the health systems of nations, particularly those of the least-developed countries, as they must now address a double burden of acute and chronic diseases amidst scarce resources.^2^-^4^ Furthermore, this epidemiological transition is adversely impacting on socio-economic development of nations, as NCDs tend to be more prevalent in young working class people.^2^ As a more sophisticated workforce becomes a highly valued and harder-to-replace economic investment, the increasing prevalence of NCD risk factors in developing countries, particularly sub-Saharan Africa (SSA), becomes a real threat to economic progress, adversely impacting on all the previous gains made in combating HIV, malaria, tuberculosis and other infectious diseases.^5^

In Uganda, while acute infectious communicable diseases still contribute the major (75%) disease burden, with malaria, acute respiratory infections and HIV/AIDS among the top 10 causes of illness and death,^6^ the burden of NCDs is increasingly posing a threat of dual epidemics of communicable and non-communicable diseases. The International Diabetes Federation put estimates of incidence of diabetes mellitus in Uganda at 50 000 affected individuals in the year 2003, and projected a 10-fold increase in the cases of diabetes by 2025 if no interventions are initiated.^7^ Estimates suggest that as many as 8% of people living in Kampala may have type 2 diabetes (T2D),^8^ while deaths attributed to NCDs in Uganda were estimated at 31 700 in 2002.^9^

Estimates of age-standardised mortality from NCDs suggest that countries in SSA, including Uganda, might have a more than three-fold higher mortality rate than several European countries, including the UK.^9^ However, these estimates are based on limited data and statistical models derived from child mortality rates and cause-specific rates from external sources. Several publications have highlighted the need for local high-quality epidemiological data on the burden of NCDs and their risk factors, particularly in SSA where such data are scarce.^10^,^11^-^14^

To date, there has been no systematic population-based study on NCD risk factors conducted in Uganda. Accordingly, between December 2011 and February 2012, we conducted a cross-sectional survey using the WHO NCD STEPS survey tools to determine the magnitude of NCDs and their risk factors in Kasese district, Uganda to serve as a pilot study for the nationwide survey of NCD risk factors.

## Methods

Ethical approval was granted by the Uganda National Council for Science and Technology’s Human Research and Ethics Committee, and the President’s Office Research Secretariat. Written informed consent was obtained before participants were enrolled in the study, using the WHO NCD STEPS survey consent form.

This study was a community population-based, cross-sectional survey designed according to a WHO STEPwise approach to chronic disease risk-factor surveillance.^15^ Data were collected in three steps; step 1 used a questionnaire to collect demographic and lifestyle data; step 2 involved measurements of height, weight, blood pressure (BP), waist and hip circumference; and step 3 used laboratory (biochemistry) investigations.

Kasese district is divided into two counties, Bukonzuo (10 sub-counties) and Busongora (12 sub-counties). One sub-county was selected from each county. Bugoye sub-county from Busongora is predominantly rural, whereas Mpondwe sub-county from Bukonzuo is peri-urban. The two sub-counties selected are the most populous in each county. Both sub-counties comprise 14 parishes, 61 villages with a total of 11 986 households. Using the cluster sampling method, seven households were randomly selected from each village. Finally, at least one adult in the selected households was invited to participate. Where a household had no consenting adults, the neighbouring household was approached.

The survey was conducted using the WHO recommended STEPwise approach.^16^ Step 1, the survey questionnaire, was administered by the field staff. It consisted of core (age, gender, education in years, current exposure to tobacco and alcohol, diet and physical activity), expanded (rural/urban setting, occupation, average household income) and optional (marital status, medical and health history, past history of smoking and alcohol consumption) variables. The medical and health history component included questions on medication, cigarette use, diabetes mellitus and hypertension.

Step 2 involved physical body measurements, including BP, height, weight, and waist and hip circumference measurements. BP measurements were taken using battery-powered digital BP machines (Omron M3-I). The participant was asked to sit on the chair and rest quietly for 15 minutes with his/her legs uncrossed. The left arm of the participant was then placed on the table with the palm facing upward. Three readings, three to five minutes apart, were then taken on the left arm. During the analysis the average of the last two readings was the final BP reading used.

Height was measured with the participant standing upright against a wall on which a height mark was made. Measurements were taken with the participant barefoot, standing with the back against the wall and head in the Frankfort position, with heels together. The participant was asked to stretch to the fullest. After being appropriately positioned, the participant was asked to exhale and a mark was made with a white chalk to mark the height. The height was then measured to the nearest 0.1 cm from the mark to the floor using a tape measure.

Weight measurements were taken on a pre-calibrated weighing scale (Seca scale). Participants were weighed dressed in light clothing and barefoot. Measurements were taken to the nearest 0.1 kg.

Step 3 involved laboratory tests. Consenting participants were asked not to consume any food, only water from after supper that day until the survey team collected the blood samples the next day (eight-hour fast). People converged at the agreed place in their community. Those who had complied with the overnight fast were eligible for finger-prick blood sample collection. Total cholesterol (TC) and triglyceride (TG) levels were measured using Reflotron-Plus machines manufactured by Roche. Fasting blood glucose (FBG) level was measured on two machines, the Accu-Chek Active glucometer from Roche and the Soft-Style glucometer from Chem-labs.

Hypertension was defined as a diastolic BP of 90 mmHg or more, or a systolic BP of 140 mmHg or more, or currently on medication for hypertension (documented in the health booklet). Diastolic BP ≥ 110 mmHg or systolic ≥ 180 mmHg was considered to be severe hypertension. Raised fasting blood glucose was defined as a blood glucose level ≥ 7.0 mmol/l or currently on medication for diabetes mellitus (documented in the health booklet). Raised total cholesterol was defined as cholesterol level ≥ 5.0 mmol/l. Overweight was defined as body mass index (BMI) ≥ 25.0 kg/m^2^ and obesity as BMI ≥ 30.0 kg/m^2^.

Excessive or harmful use of alcohol was defined as the consumption of five or more for men, four or more for women, standard units per day for three or more days per week. Physical activity was measured using questions on four different aspects: physical activity at the workplace, physical activity during recreation time, physical activity while travelling, and physical resting time. A heavy smoker is, according to the recommendations of the World Health Organisation (WHO), a smoker with a daily consumption of more than 20 cigarettes.

## Statistical analysis

Data were collected manually using case record forms (CRFs), captured into epi-data and later transferred to STATA version 10 for analysis. Values are expressed as percentages of total respondents. Simple bivariate analysis was used to analyse the data. Priority was given to practical benefit and clinical significance in interpreting statistically significant data. Statistical significance was set at *p* < 0.05.

## Results

**Fig. 1.**
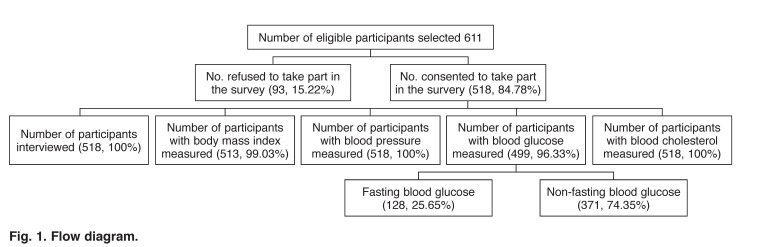
Flow diagram.

A total of 611 eligible adults were selected and approached to participate in the survey. Of these, 93 (15.22%) refused while 518 (84.87%) consented to take part in the survey. Of the 518 participants who took part, 56% were female and 29% had no formal education, while 41% had primary school education. BP, and fasting blood sugar and total cholesterol levels were measured in 100, 25.7 and 27.8%, respectively of the 518 participants (Fig. 1, Table 1).

**Table 1:** Characteristics Of The Study Participants

		*Male*		*Female*	
	*Total*	*n*	*%*	*n*	*%*
Gender*	528	297	45.5	231	54.5
Age (years)					
25–34	179	98	34.0	81	35.1
35–44	118	63	21.9	55	23.9
45–54	104	57	19.9	47	20.2
55–64	57	36	12.8	21	9.3
> 64	60	33	11.5	27	11.5
Education					
None	112	41	14.1	71	30.9
Primary school	243	129	45.1	114	49.7
Secondary school (O level)	113	82	28.3	31	13.5
Secondary school (A level)	27	18	6.4	9	3.9
University/college	23	17	6.1	6	2.0
Occupation					
Peasant	326	159	55.6	167	72.2
Trader	20	11	3.7	9	3.9
Teacher	28	22	7.4	6	2.5
Housewife/homemaker	10	0	0	10	4.49
Other	80	66	23.3	14	6.2
None	54	29	10.1	25	10.7
Marital status					
Married	411	244	84.9	167	71.9
Separated	38	17	6.1	21	9.3
Widowed	39	8	2.4	31	13.5
Never married	30	18	6.7	12	5.3

*Percentage is by column for gender only. The rest of the variables are by rows.

**Fig. 2.**
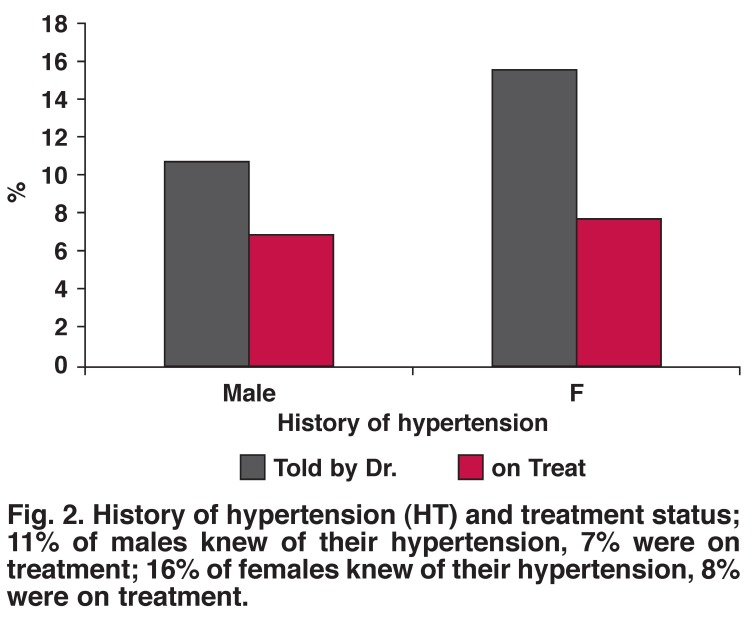
History of hypertension (HT) and treatment status; 11% of males knew of their hypertension, 7% were on treatment; 16% of females knew of their hypertension, 8% were on treatment.

**Fig. 3.**
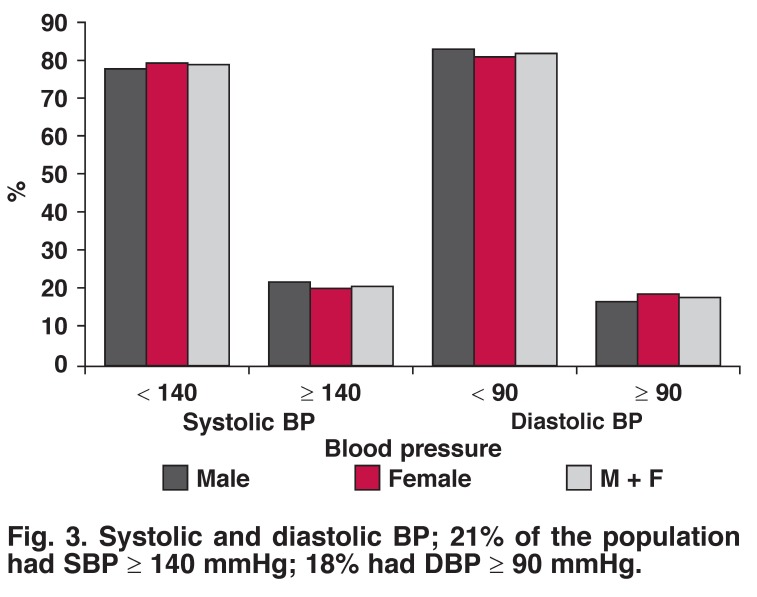
Systolic and diastolic BP; 21% of the population had SBP ≥ 140 mmHg; 18% had DBP ≥ 90 mmHg.

Sixteen per cent of females and 11% of males were told by the doctor that they had hypertension, while only 8% of females and 7% of males with diagnosed hypertension were currently on medication for hypertension (Fig. 2). Twenty per cent of females had SBP ≥ 140 mmHg and 20% had DBP ≥ 90 mmHg; while 22% of males had SBP ≥ 140 mmHg and 17% had DBP ≥ 90 mmHg (Fig. 3). There was no statistical difference between the genders (SBP, *p* = 0.758; DBP, *p* = 0.503).

**Fig. 4.**
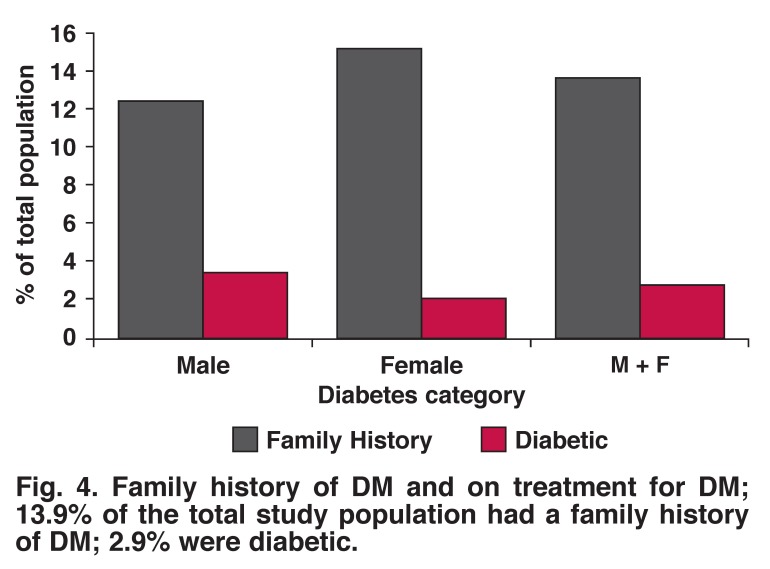
Family history of DM and on treatment for DM; 13.9% of the total study population had a family history of DM; 2.9% were diabetic.

**Fig. 5.**
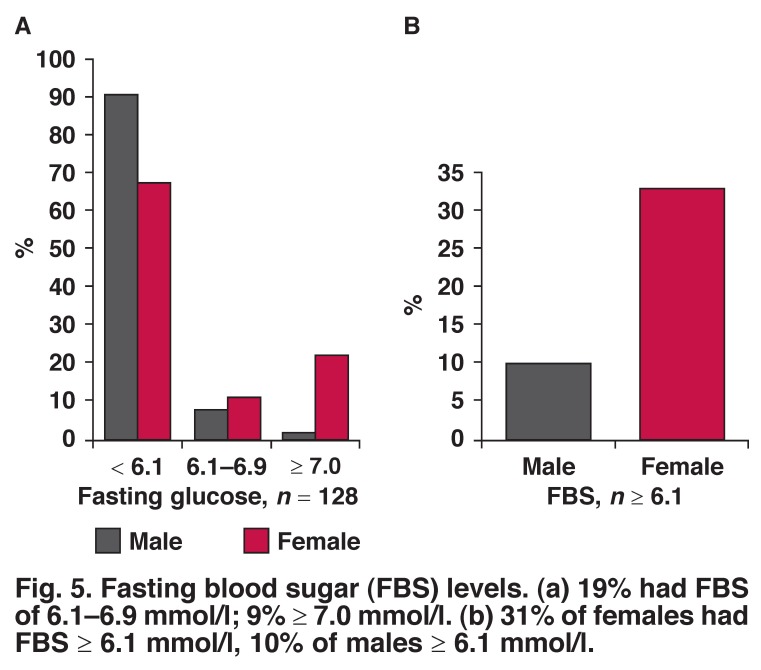
Fasting blood sugar (FBS) levels. (a) 19% had FBS of 6.1–6.9 mmol/l; 9% ≥ 7.0 mmol/l. (b) 31% of females had FBS ≥ 6.1 mmol/l, 10% of males ≥ 6.1 mmol/l.

Ten per cent of males had fasting blood sugar levels > 6.0 mmol/l compared to 33% of females, while 12.5% of male had a positive family history of diabetes mellitus (DM) and 3.5% were on treatment for DM. Sixteen per cent of females had a positive family history of DM and 2.2% were on treatment for DM (Figs 4, 5).

**Fig. 6.**
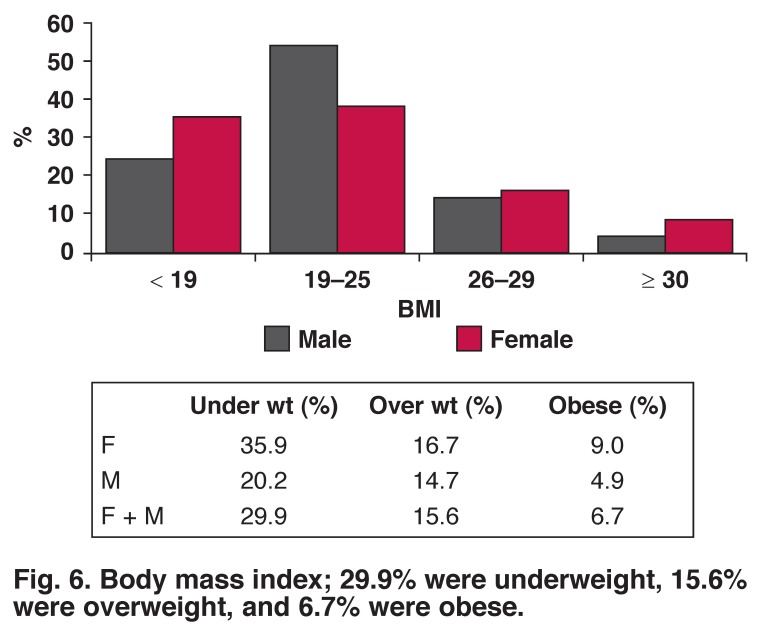
Body mass index; 29.9% were underweight, 15.6% were overweight, and 6.7% were obese.

**Fig. 7.**
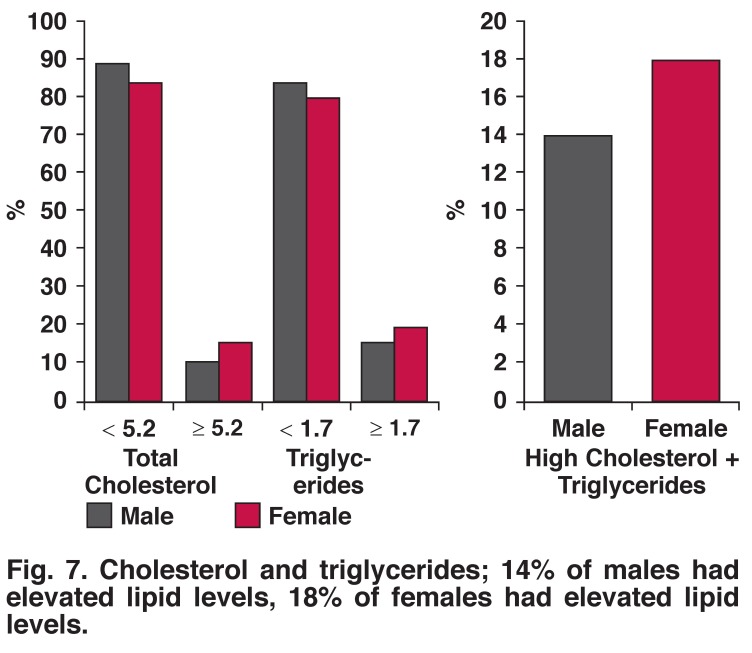
Cholesterol and triglycerides; 14% of males had elevated lipid levels, 18% of females had elevated lipid levels.

The prevalence of underweight, overweight and obesity were more frequent in women than men (35.9 vs 20.2%, 16.7 vs 14.7% and 9.0 vs 4.9%) (Fig. 6). Raised total cholesterol was more frequent in women than men (16 vs 11%). Ten per cent of the population had elevated total cholesterol levels while 21% had elevated triglyceride levels (Fig. 7). Nine per cent of males and 5.7% females ate five or more servings of fruit per day; 1.2% of males and 1.1% of females ate five or more servings of vegetables per day.

**Fig. 8.**
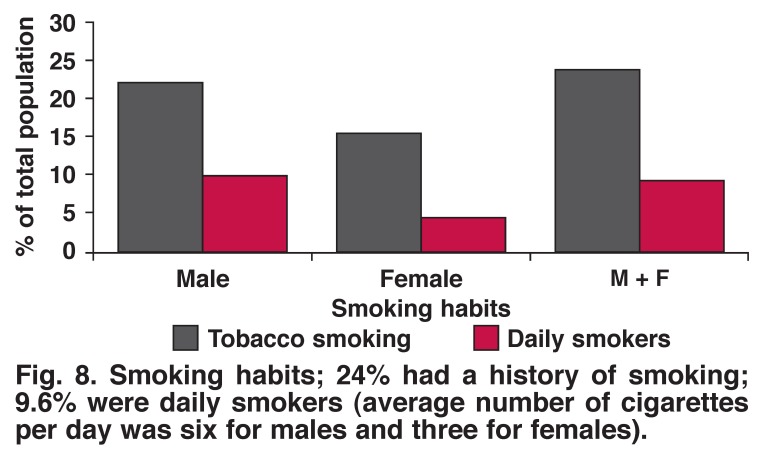
Smoking habits; 24% had a history of smoking; 9.6% were daily smokers (average number of cigarettes per day was six for males and three for females).

**Fig. 9.**
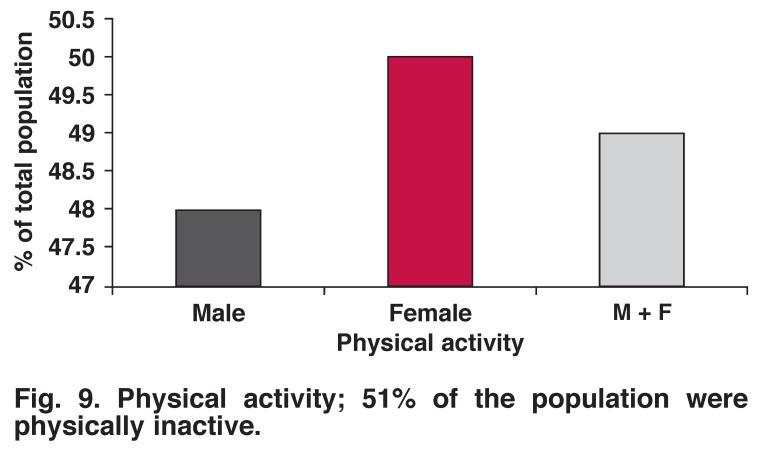
Physical activity; 51% of the population were physically inactive.

**Fig. 10.**
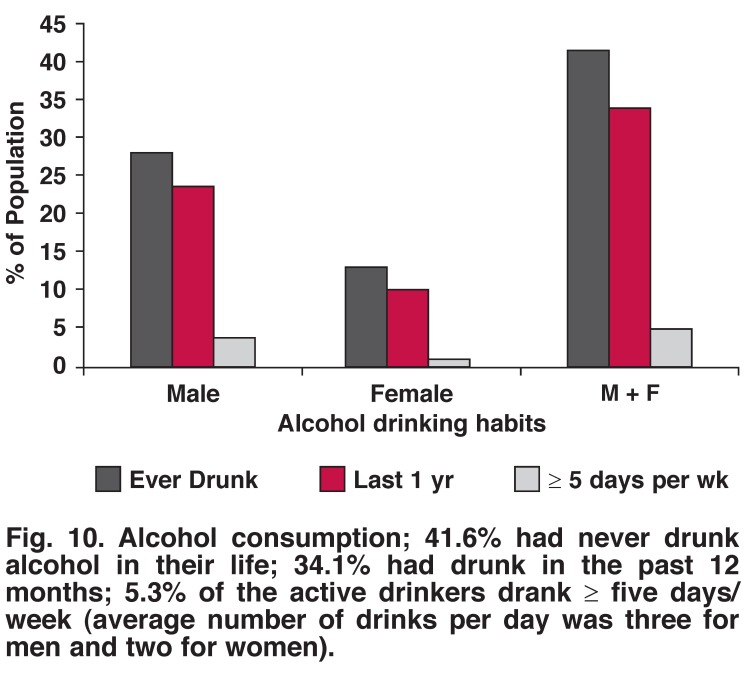
Alcohol consumption; 41.6% had never drunk alcohol in their life; 34.1% had drunk in the past 12 months; 5.3% of the active drinkers drank ≥ five days/week (average number of drinks per day was three for men and two for women).

**Fig. 11.**
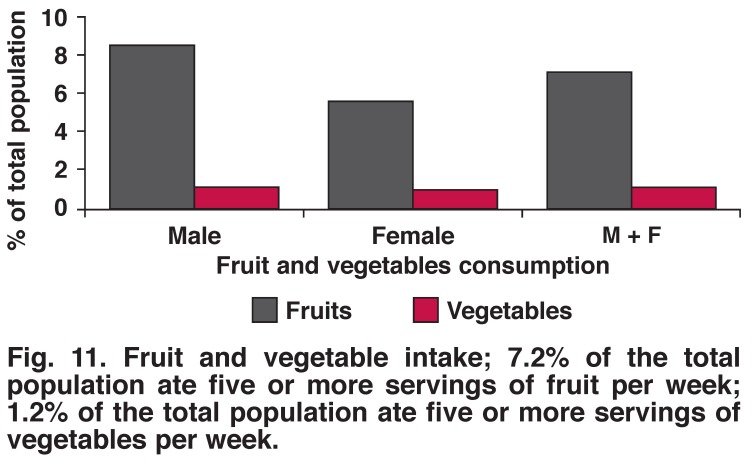
Fruit and vegetable intake; 7.2% of the total population ate five or more servings of fruit per week; 1.2% of the total population ate five or more servings of vegetables per week.

Tobacco smoking, alcohol drinking (any amount) and excessive alcohol drinking were more common in men than women (22.5 vs 15.5%, 23.9 vs 10.3%, 4.1 vs 1.2%, respectively). There was no significant difference between the genders with regard to physical activity (52% male, 50% female, *p* = 0.703) (Figs 8, 9, 10, 11).

## Discussion

This is the first population-based survey using internationally standardised protocols to report the prevalence of risk factors for NCDs in the Kasese district of Uganda. This study demonstrated that chronic non-communicable diseases and their risk factors constitute a public health problem in the Kasese district, with at least one in five men smoking tobacco, one in five with hypertension, one in 10 with a positive family history of DM, one in five being pre-diabetic and therefore a candidate for the metabolic syndrome, and one in five overweight/obese.

The first major finding of this study was the high prevalence of hypertension, both self-reported and point-measured BP during the survey. The majority of people with hypertension did not know they had this medical problem, which is consistent with findings from other studies in sub-Saharan Africa.^17^ Hypertension is the leading cause of stroke in Africa. A further finding that only 3.7% were on treatment reflects the low level of knowledge of the dangers of untreated hypertension in the population. A striking finding was that there was no difference in the prevalence of hypertension between the genders.

Factors not measured in this survey, and which may explain the observed risk among women, were hormonal status, saturated fat consumption and salt intake. Also, further studies should be done to document the proportion of those on treatment whose BP is under control, as well as the presence of hypertensive heart disease among those with hypertension.

The second major finding was that the risk profile of this predominantly rural population of Kasese was markedly different from that reported previously for the urban and peri-urban settings.^10^-^12^ The prevalence of raised blood glucose levels (defined as capillary whole BG of at least 6.1 mmol/l) in 31% of females and 10% of males, DM (13.9% had a family history of DM, 2.9% were diabetic), raised BMI (15.6% were overweight, 6.7% were obese), and tobacco smoking (24% had history of smoking with 9.6% heavy smokers) were markedly higher than previously speculated. The level of physical activity was surprisingly lower than expected in this predominantly hilly area, although different definitions of physical activity could have led to this response. These findings support the need for regular screening of individuals for NCDs and their risk factors.

There was a high prevalence of underweight people (29.9%). When taken together with the observed rates of DM and glucose intolerance, questions arise with regard to the possibility of a connection between under-nutrition and DM.

In general, this study highlights the need to undertake population-based studies in all districts in the country to quantify the magnitude of NCDs at a national level. It is evident that there is variation among ethnic groups and locations, as various factors contribute to the development of disease and other factors contribute to the perpetuation of diseases.

In order to institute a cost-effective intervention, the specific factors at play in a given population must be identified. It may not be appropriate to generalise these findings to refer to the Karamoja population. These results though are useful in guiding intervention and preventative measures for the Kasese population, and should be well received by policy makers in the local government of Kasese, as well as the ministry headquarters. For example, vegetables and fruits are grown in large quantities in Kasese, but consumption is low. Most are sold to the cities. The population is not aware of the benefits to their health of eating fruit and vegetables. Mass education to encourage increased consumption of fruits and vegetables will benefit the population.

A key strength of this study was the use of a representative sample, with analysis taking into account the complex survey design. The relatively high response level minimises the likelihood of selection bias, and the range of factors that were measured should be a good reflection of those factors in the Kasese population. The use of WHO standardised protocols, intensive training of data-collection staff, pre-study testing of procedures, and the close supervision of staff during data collection all highlight the attention that was paid to minimising avoidable sources of measurement error.

Limitations of this study need to be borne in mind. The STEPS methodology is designed to provide standardised information on key modifiable risk factors that can be measured in population-based surveys without resorting to high-technology instruments. It is not designed to measure total energy intake, dietary fat, dietary sodium, body fatness or physical activity by objective methods, such as accelerometry and pedometry. Information on these factors would have provided a more comprehensive picture of the relationships we studied. In addition, these cross-sectional data do not allow age-related differences in BP, blood glucose and total cholesterol levels to be attributed to ageing, independent of cohort effects. Assessment of risk factors by age group as well as fasting blood sugar level for different BMIs would have provided more insight. Finally, due to lack of power, we were not able to assess the relationship between underweight and diabetes.

## Conclusion

This study provides the first NCD risk-factor profile of people in the Kasese district, Uganda, using internationally standardised methodology. Our findings for this predominantly rural sample provide evidence for health policy-makers as well as district authorities on lifestyle problems in the population studied. The burden of more diseases is to be expected if an effective prevention strategy is not undertaken.

Although even short-term educational programmes have been shown to be effective in improving lifestyle, a durable education strategy and cost-saving policies supported by sustained largescale media education and school-based educational programmes could be the starting point for a possible national programme on controlling NCDs in Uganda. A national NCD risk-factor survey should however be undertaken to avoid biased generalisation of results, as Kasese is not a representative population of Uganda.
